# Social inclusion of persons with disability in employment: what would it take to socially support employed persons with disability in the labor market?

**DOI:** 10.3389/fresc.2023.1125129

**Published:** 2023-06-30

**Authors:** Ivy Chumo, Caroline Kabaria, Blessing Mberu

**Affiliations:** Urbanization and Wellbeing, African Population and Health Research Center (APHRC), Nairobi, Kenya

**Keywords:** social inclusion, persons with disability (PWDs), informal settlements, employment, sustainable development

## Abstract

**Introduction:**

One of the major challenges that persons with disabilities (PWDs) are facing globally is unemployment. The challenge is attributed to systems that are not built with inclusivity in mind by employers. As such, the work of inclusion is not inviting PWDs to do more but to make a difference through social support. Most research on inclusion in the employment of PWDs in low-income settings has been concentrated upon the labor “supply” side, and to the best of our knowledge, no specific studies moved toward inclusion in employment issues from the employers’ perspective in informal settlements. Notably, our research question is: “what would it take to socially support employed PWD in informal settlements building from the perspectives of employers.”

**Methods:**

This paper used data from in-depth interviews with 38 service providers in the education, health, water, sanitation, and solid waste management sectors and two sub-county officials in two informal settlements in Nairobi, Kenya. The service providers were employers or entrepreneurs who had hired PWDs in their workspaces and the sub-county officials that had vast experiences with employed PWDs. Data from transcripts were analyzed by the research team using content analysis.

**Results:**

The social support offered to employed PWDs included listening to them with a concern; identifying their strengths and obstacles; planning for them based on their qualities, knowledge, and experience and linking them with existing opportunities; creating specific opportunities and facilitating their access to opportunities; gradual withdrawal of support by support group; and, lastly, compromise by employers with PWD inclusion strategies. Study participants described how misdirected and inadequate resources, dissatisfaction and unhappiness, and conflicts at the workplace associated with non-inclusion were constraints to social support. Employment matters affecting PWDs are complex and require multi-pronged context-specific social support approaches. Essential to the functioning of an inclusive workplace for PWDs were communication, coordination, sharing of the workload, and supporting individual PWD.

**Conclusion:**

Inclusion of PWDs in the labor market is about generating a supportive workplace where people are valued and appreciated without judgement for what they can contribute. Notably, in the absence of jobs for everyone and high unemployment rates among every segment of the population, there is a need for an awareness creation, mobilization, and sensitization of employers and investors around the competencies of PWDs and their need to socially support on an impartial basis. On the other hand, employment centers could establish stations in low-income areas to advise and support PWDs on career opportunities that are disability-friendly and partner with employers to avail information about the capabilities of PWDs. Conversely, the government should provide some tax-related benefits to employers to upsurge employer incentives for hiring PWDs and empower employers on benefits and positive culture of employing PWDs. At all times, employers should be hands-on and involve diverse stakeholders to implement current policies and frameworks in different work contexts across the country and beyond.

## 1. Introduction

The Sustainable Development Goals 2030 agenda and its 17 goals offer a framework to guide local and international communities toward the accomplishment of a disability-inclusive progress (1). It pledges to leave nobody behind, including persons with disabilities (PWDs), and has acknowledged disability as a cross-cutting concern to be considered in the execution of all the 17 goals (2). Similarly, it recognizes that vulnerable groups including PWDs must be empowered in all spheres of life including in the labor market (1, 2). PWDs include individuals who have long-standing impairments (i.e., physical, mental, intellectual, or sensory), which interacts with diverse barriers, hence hindering complete and active participation on an equivalent basis with others (3, 4). Employers are obliged to ensure complete, active, and equal enjoyment of basic human rights and autonomy in the labor market for economic balance (5). Sustainable Development Goal 8.5 specifically targets to achieve “full and productive employment and decent work for all,” including PWDs by 2030 (1).

Key actors have made efforts both at local and international spheres to upsurge the participation of PWDs in the labor market regarding employment (6). Employment is not only a vital idea in economics but also a crucial element in realizing a good standard of living, as it offers an opportunity for self-reliance (7), dignity (8), and a means of individualism (7). Greater participation of PWDs in labor market can increase their social inclusion (7), thus a key step to their enablement, liberation, and wellbeing (7). It is in this regard that Article 27 of the Convention on the Rights of Persons with Disabilities (CRPD) emphasizes unrestricted right of entry to labor market for PWDs. The article requires that the state-owned parties embark on actions to outlaw all forms of discrimination and to make a conducive and just environment for PWDs at work, while ensuring a professional growth similar to a person without a disability (3). The employment of PWDs has always been a challenge in Kenya; however, the current paradigm shift after the country co-hosted the Global Disability Summit in 2018 has gained traction over other low- and middle-income counties. The commitments on the summit were focused on PWDs, their rights, and their environments, with a sense of direction, purpose, and understanding (9), so as to eradicate poverty. Poverty among PWDs cannot be eradicated easily if they continue depending on their caregivers for financial empowerment. As such, the government and other relevant actors should endeavor to create a supporting environment (i.e., that enforces current laws, avails incentives to employers of PWDs, and actors who offers skills for PWDs) to motivate employers’ obligation to employ PWDs (6). Some informal settlements in Kenya are accommodative of PWDs, due to socialist nature of residents and the existence of support structures (10, 11). Informal settlements (i.e., unplanned sites that are not compliant with authorized regulations) are characterized by residents seeking available opportunities (12). Notably, there are few formal opportunities (13), and residents end up relying on the private and informal sectors for labor (14, 15). Under such circumstances, many residents have unmet needs (16), therefore leaving communities including PWDs to find ways of fulfilling their own needs by seeking employment opportunities. Due to vulnerabilities of PWDs (17), some employers accommodate them in the workplace; yet, no study has uncovered social support of PWDs in informal settlements from the employers’ perspectives.

As Kenya grapples with socioeconomic development due to repercussion of the COVID-19 pandemic, employers must consider the inclusion of PWDs in the new forms of work and industries that have emerged (6, 18). Despite the significance of employment and the right of everyone to access decent jobs without discrimination (18, 19), employed PWDs face significant social support-related challenges (20). Approximately 25% of adults live with a disability (i.e., cognitive, physical, or emotional disability, among others), and many of them have talents (21). Therefore, the task during inclusion is not inviting PWDs to do extra, but to create an accommodative change in the environs that we invite them into (4, 19). The challenge does not lie with a PWD, but with systems that are not built with inclusivity in mind by employers or owners (22). Most research on labor, inclusion, and discrimination has been concentrated upon the labor “supply” side of the employment equivalence (i.e., obstacles, limitations, and contests in view of PWDs seeking employment or being employed) (23, 24), and to our knowledge, no specific studies in low- and middle-income countries (LMICs) loomed these employment and inclusion issues from the employers’ perspective (20), more so in the informal settlements. Could insights from the employer's side be a resolution to this ostensible challenge? And, has anyone asked employers and entrepreneurs as they are “missing voices”? Concurrently, actors for various justice groups highlighted that some PWDs are ready, willing, and able to work but quit their job once recruited due to lack of support at the workplace (20).

Beyond the lack of consultation on suitable information (i.e., on low-cost technology preferences, inclusive design and its cost, capacity building, and behavior change) (25), less has been uncovered concerning the “software” features of service delivery (i.e., changes needed for organizations to be inclusive for all workers and changes in how programs are planned and executed) (18, 25). To this end, we acknowledge that there are many aspects to the employers’ side. First, there is the desire and willingness to address the issue of inclusion. Second, there is how to go about support systems, and, third, there are policies (and funding) for workplace accommodations and flexibility in the design of work tasks and work scheduling. In this study, we focus on the second aspect: social support pathways from the employer’s perspectives. We involved service providers who had employed PWDs to uncover available social support that sustained the engagement of PWDs in their specific workplaces and documented their best practices and challenges in creating inclusive work environments. As such, our research question is: “what would it take to socially support employed PWD in informal settlements building from the perspectives of employers?”

### 1.1. Theoretical background and conceptual framework

**Figure 1:**
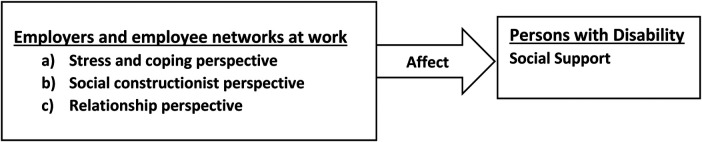
Conceptual framework.

We embed this study on the social support theory by Cullen (26, 27). Cullen distinguished between macro-level and interpersonal-level effects of support, emphasizing how supportive societies and supportive relationships can enhance safe and effective working (27). Social support is commonly conceptualized as the social resources on which an individual can rely on when dealing with lived challenges, realities, and stressors. Three important theoretical perspectives on social support research are (a) the stress and coping perspective, (b) social constructionist perspective, and (c) relationship perspective. The stress and coping perspective proposes that support contributes to a person's life by protecting them from the adverse effects of stress. The social constructionist perspective proposes that support directly influences a person's life by promoting self-esteem. On the other hand, the relationship perspective proposes that social support cannot be separated from relationship processes that often co-occur with support, such as creating links, networking and opportunities and low social conflict (28). Social support mechanisms ought to be qualified by the fact that many different interpersonal processes and constructs have been included under the rubric of social support (29) (Figure 1). Unlike other societal related theories, social support theory focuses on how something positive can prevent or reduce risks (26, 30).

The theory is applicable to our study because PWDs need social support for their effective functioning. Notably, they often receive negative treatment such as being labelled based on their disability status (31) and are vulnerable to a greater risk of becoming victims of crime (17) and social and psychological problems (31). PWDs have also been perceived as dependent, incompetent, unproductive, ill, burdensome, unattractive, hypersensitive, helpless, passive, and childish, and as such, they have many unmet needs (16). For this reason, some employers have considered their talents and employed them at their workplace. From our position, we believe that there is a need for social support to employed PWDs at the workplace, even when they have talents, more so in the informal settlements where government social rehabilitation services are inexistent or inadequate (17, 32). Despite the talents and the fortune of being employed, PWDs often condemn and hurt themselves since they cannot accept their imperfection and often feel ashamed, anxious, incapable, unfortunate, miserable, inattentive, and meaningless (31). These conditions indicate that their self-esteem tends to be low. Self-esteem determines personality and affects the human health state and effective functioning at work (16).

## 2. Methodology

We present our study findings according to a set of standardized Consolidated Criteria for Reporting Qualitative Research (COREQ) (33).

### 2.1. Study objectives and design

We document lessons on what it takes to socially support an employed PWD in informal settlements, from the perspectives of employers and entrepreneurs. This was a qualitative study using in-depth interviews (IDIs) (33). We designed the study guided by social support theory. As such, questions in the study tools probed on social support strategies by employers to employed persons with disability. The social support theory formed a basis for our objectives, research questions, data collection, analysis, and interpretation of findings.

Beyond reported challenges faced by PWDs relating to negative treatments and associated psychological problems of self-esteem and other stressors in the community and at work, our study, guided by the social support theory, especially the postulation that social resources can be relied upon by individuals when dealing with lived challenges, realities, and stressors, builds on the need to understand the pathways through which PWDs will obtain the social resources they need for their effective functioning in their workplace. We sought to document what it will take to socially support an employed PWD from the perspectives of employers and entrepreneurs. This objective and theoretical orientation informed our deployment of the IDI methodology, data collection, data analysis, and ethical procedures implemented in our study. These spectra of study tools deeply probed into questions that identified and highlighted specific strategies engaged by employers to address the needs of PWDs working in their organizations. IDIs are veritable qualitative tools for direct, one-on-one engagement with principal actors in a study. Specifically, in our case, IDIs pinpoint not only the social support needed but also the associated challenges in their implementation as well as how to sustain such supports in the service of unmet needs of their employees with disabilities.

### 2.2. Study setting

**Figure 2:**
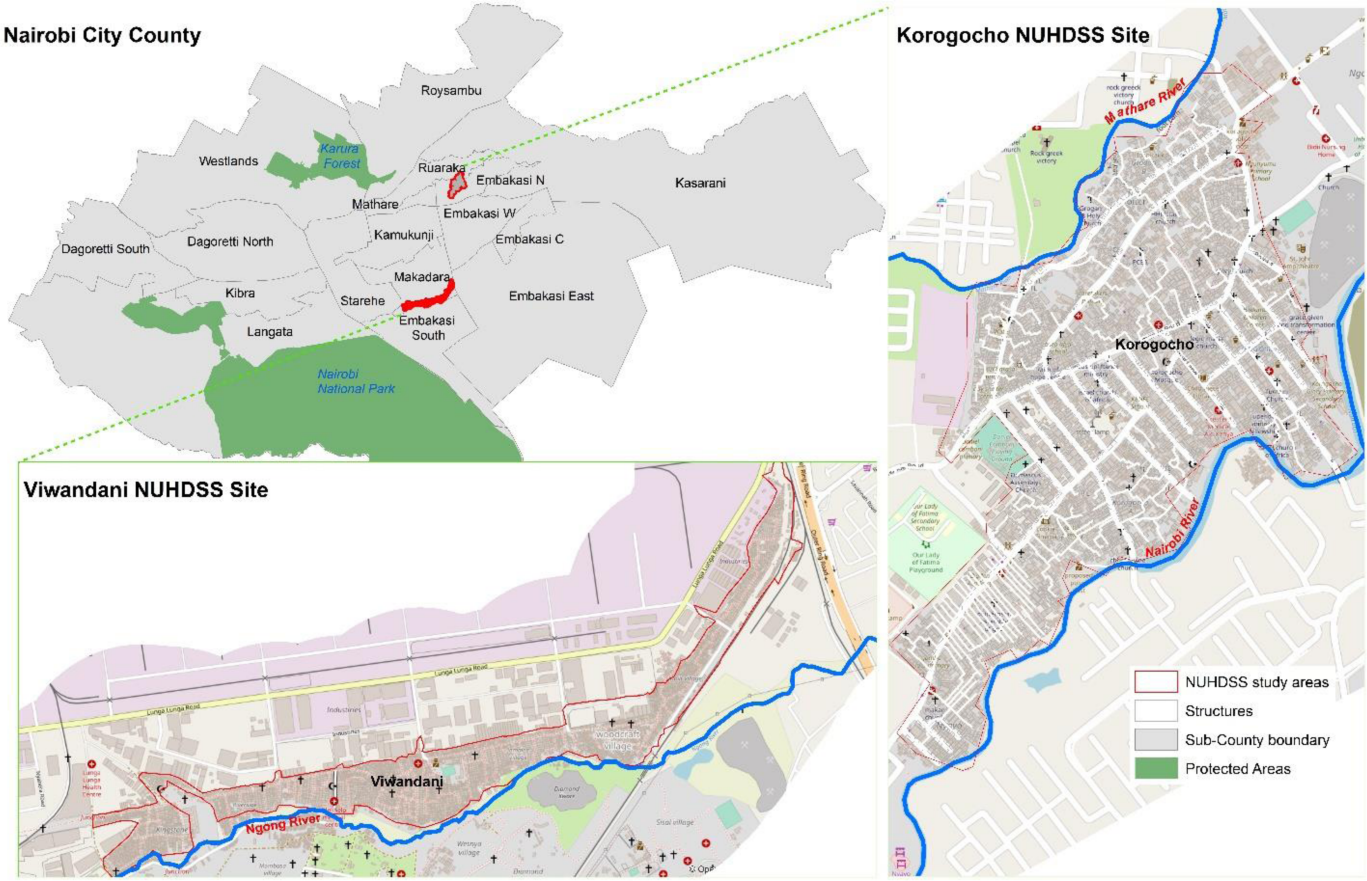
Study sites (source: authors 2022).

From a population of 350,000 in the 1962 census to 4,397,073 in the 2019 census, Nairobi typifies the rapid urbanization and population explosion in sub-Saharan Africa (34). As the capital and largest city of Kenya, Nairobi has always been the major attraction of various segments of the Kenyan population—in search of better livelihood opportunities, including the marginalized and vulnerable groups such as persons with disability (16). The consequence of the rapid and uncontrolled population explosion is the proliferation of informal settlements in Nairobi, with upwards of 60% of Nairobi residents estimated to be living in slums and contributing to increasing urbanization and the need for employment opportunities. Our study covered two informal settlements of Korogocho and Viwandani informal settlements in Nairobi, in the regions covered by Nairobi Urban Health and Demographic Surveillance System (NUHDSS) initiated in 2002 by the African Population and Health Research Center (APHRC) (35) (Figure 2). Informal settlements of Korogocho have a more steady population, and multi-generational inhabitants reside in the region for several years (36). On the other hand, Viwandani is situated nearby an industrial zone with many vastly traveling and more educated occupants who labor or look out for better jobs outside the informal settlements (36).

### 2.3. Target population, sampling, and sample size

The population of interest were service providers in five sectors that were identified and described in the governance diaries as priority basic needs (16). The sectors included education, health, water, sanitation, and solid waste management. Through a stakeholder mapping exercise, we developed a stakeholder database that depicts actors/service providers with employed PWDs and were re-defined and confirmed by PWDs interviewed during governance diaries data collection process (13) and during community advisory study consultation (10). From the service providers identified, we purposively selected four education providers, two healthcare providers, four water providers, four sanitation providers, and four solid waste management providers in each of the study sites. Two sub-county officials in each study site were also purposively selected if they had experiences in offering social support to employed PWDs in the sub-county. This enabled us to understand the social support offered to employed PWDs, challenges faced in social support, and how to maintain the support.

### 2.4. Data collection process

We collected data from March to May 2022. We selected Research Assistants with support from community advisory committees (10), if they were endorsed by community leaders in the study sites and if they had some experience in qualitative research. We opted for Research Assistants from the community who are insiders. Insider researchers are native to the setting and are usually perceived to be too close, thereby not attaining the distance and objectivity necessary for valid research (37). Notably, we challenged the limitations of using insider Research Assistants in this study. Insider knowledge in whatever research tradition is not only valid and useful but also provides important knowledge, in which approaches used by outsiders may not be able to uncover (38). In our view, insider research is not problematic in itself and is a respectable research in whatever paradigm it is undertaken. Data were collected through IDIs (33), using an IDI guide which had questions on social support available for employed PWDs at the workplace. We conducted face-to-face interviews in English or in Swahili, at a quiet location convenient to the participants which were mainly at their workplace and in convenience of the study participants. Research Assistants recorded the interviews using a digital recorder and backed up with handwritten records. These interviews lasted for approximately 1 h. The sampling of study participants for the interviews continued until no new information was forthcoming.

### 2.5. Data quality control

Project Researchers reviewed all audio files on real time to ensure completeness and depth of the interviews and provided feedback to the Research Assistants, who were trained for 5 days on study aims, data collection procedures, study tools, and study ethics. Researchers and Research Assistants held debriefing sessions every day to determine the key emerging themes, probing techniques, and general progress.

### 2.6. Data management and analysis

Recorded audios from IDIs were translated and transcribed from Swahili to English and saved as individual Microsoft Word documents. Outputs were assigned number codes to prepare for analysis and to ensure confidentiality. Thereafter, transcripts were imported into NVivo 12 software (QSR International, Australia) for coding and analysis. Each transcript had a unique identifier comprising participant category, study site, and sex to enhance anonymity and facilitate informed analysis.

We used a framework analysis (39), informed by the social support theory (28) (Figure 1). Framework analysis is adopted for research that has specific questions, a pre-designed sample, and priory issues (39)^.^ The first step of framework analysis was listening to the recordings to familiarize the researchers with the information related to social support, challenges, and maintenance of social support. To ensure reliability, two researchers (an experienced qualitative researcher with experience in labor markets and an anthropologist) and five co-researchers, who collected the data participated in the development of a coding framework by reading the outputs imported in NVivo 12 software independently to establish an inter-coder agreement. Once the initial coding framework was completed, the team met to discuss the themes generated and to reach an agreement on themes (Table 2). Two researchers proceeded with coding, charting, mapping, and interpretation of transcripts.

### 2.7. Ethical considerations

AMREF Health Africa's Ethics & Scientific Review Committee (ESRC) (REF: AMREF-ESRC P747/2020) approved the study. We obtained a research permit from the National Commission for Science, Technology and Innovation (NACOSTI) (REF: NACOSTI/P/20/7726). Approval was also sought from the Liverpool School of Tropical Medicine (LSTM) and the APHRC internal ethical review committees as part of the larger Accountability and Responsiveness in Informal Settlements for Equity (ARISE) Hub funded by the UK Research and Innovation (UKRI). Before participating in an interview, all participants provided an informed written consent. The interviews were conducted in quiet spaces for privacy and confidentiality and for the quality of the audio files.

## 3. Results

We present findings on social support for PWDs, challenges faced by social support, and maintenance of support. Study participants included employers or service providers who had recruited PWDs in the workplaces and sub-county officials with lived experiences on employment of PWDs in the two study sites. Specifically, four education providers, two healthcare providers, four water providers, four sanitation providers, four solid waste management providers, and two sub-county officials in each of the study sites took part in our study (Table 1).

**Table 1:** Summary of the study's sample coverage.

Study participants	Korogocho	Viwandani
Education service providers	4	4
Health service providers	2	2
Water service providers	4	4
Sanitation service providers	4	4
Solid waste management service providers	4	4
Sub-county official	2	2
Sub-total	20	20
Total	40	

The study themes and sub-themes were anchored on social support theory (Table 2). In addition, we reported emerging themes on challenges and maintenance of support.

**Table 2:** Themes of the study.

Major themes	Sub-themes
Social support	a) Stress and coping perspective • Listening with a concern • Gradual withdraw of support offered to PWDs b) Social constructionist perspective • Identifying strengths and obstacles for PWDs • Compromising of principles by support members for the benefit of PWDs c) Relationship perspective • Planning for PWDs based on their experiences • Linking PWDs with existing opportunities and creating opportunities at work
Challenges in social support	• Misdirected and inadequate resources • Dissatisfaction and unhappiness • Conflicts
Maintenance of the social support	• Communicating information • Coordinating the network • Sharing the workload • Supporting individual members

### 3.1. Theme 1: social support

Social support is commonly conceptualized as the social resources on which an individual can rely when dealing with life problems and stressors. Three important theoretical perspectives on social support research are (a) the stress and coping perspective, (b) the social constructionist perspective, and (c) the relationship perspective (Figure 1). We identified social support approaches that include a PWD being listened to with a concern; identifying strengths of a PWD and the obstacles they face; planning for the PWD based on qualities, knowledge, and experience and linking them with existing opportunities; creating targeted opportunities and facilitating access to the opportunities; the gradual withdrawal of support by the employee support team; and lastly compromise by the employer and employees as discussed below.

#### 3.1.1. Stress and coping perspective

Stress and coping perspectives include listening with a concern and gradual withdraw of support offered to PWDs, as the two strategies reduce stress.

##### 3.1.1.1. Listening to PWDs with a concern

Employers and employees without disability acted as support team/group to employed PWDs. The support groups consulted with PWDs to identify choices, roles, interests, and needs on which planning could be based. Careful listening clarified the person's choices and reasons behind choices. Communication could take place within a formal meeting or as an exchange between two people during a chance social encounter. The question may have concerned a decision as major as relocating a water point or as minor as a choice of time to arrive and leave the workplace.

*During a discussion about a person with disability attempts of relocating a water point, other employees suddenly turned to her and asked, ‘is this really what you want to do?’ Another member added ‘just be open’. The person thought for a while and then said it was what she wanted to do but didn’t know how to go about it.* (IDI, Male Water Provider, Viwandani)

The support team of employees with support from employers noticed a growth in assertiveness and confidence of employed PWDs who were listened to with a concern. Gaining input from a PWD continued to be a top priority in planning to support employees, who were always keen and alert on signs that indicated meaning and choices for PWDs.

*Despite her disability, the person has always contributed a lot to the group. We are very observant of non-verbal cues to pick on aspects to help the person work independently … we are interested in empowering them on the same.* (IDI, Female Healthcare Provider, Korogocho)

##### 3.1.1.2. Support team getting out of the way and gradual withdrawal of support

Support employee team “got out of the way” when it became obvious that the PWD was becoming more autonomous. In many cases the support team often seemed surprised and delighted at the speed and confidence with which the PWD adapted to new situations and revealed previously unnoticed strengths. Support members would then adjust and change their actions accordingly, and overtime withdraw support, as the PWD became proficient. This strategy eased transition and increased success, making participation more comfortable for the person.

*When a support group in the employment space thought the PWD is confident to execute their tasks, the support group move aside for them to work independently.* (IDI, Female Solid Waste Management Provider, Korogocho)

* When the PWD began attending soccer and counselling sessions, a support employee talked to the staff and accompanied them for support … bit by bit we wean off to a point where the PWD would book their appointment and go on their own.* (IDI, Female Education Provider, Viwandani)

#### 3.1.2. Social constructionist perspective

Social constructionist perspectives include identifying strengths and obstacles for PWDs and compromising of principles by support members for the benefit of PWDs, as the strategies enhance self-esteem of PWDs.

##### 3.1.2.1. Identifying strengths and recognizing obstacles

Employers worked on identifying the skills, talents, and personal qualities of PWDs. On some occasions where the employers and employee support groups identified a PWD as an excellent social organizer and able to connect peers to social groups and supply information about social activities, she/he was nominated as a team leader. This boosted their self-esteem as they felt recognized and supported from support group members or the employer. As such, at the time of study, an individual with disability was working with the communication team, and the communication coordinator had complemented their excellent job on packaging inclusive messages. In some cases, former life experiences such as vilification or abuse, or communication barriers, created obstacles to participation for the PWD, and the employee support teams did their best to identify and troubleshoot the obstacles.

*She loves organizing things. As such she was appointed as a team leader for an event. The members also supported her in some ways during the event … But the key thing here is that she was leading the role and her self-esteem was boosted.* (IDI, Male Education Provider, Viwandani)

*At the session, attendees include people with hearing impairments. The PWD helped with developing inclusive messages… You also find that the PWD has gone through many other painful life experiences and you have to pinpoint the challenges as you would not want to add more pain at the workplace.* (IDI, Male Healthcare Provider, Viwandani)

##### 3.1.2.2. Compromise by employers or by PWD support group

Occasionally the person and the employer or support networks held differing views. The employer or support staff may have felt that a goal was out of reach, or saw another goal as crucial, but the person held the opposite view. Discussion and compromise were required when these dilemmas were portrayed. This could be stressful for some employers or support staff. Support networks at work felt strongly enough to hold out against criticism from outside and were convinced that in specific situations they knew what was right for the person despite resistance. One person liked to dress casually even during official meetings. A network member felt that this was not appropriate, consulted with the person, and persuaded her to change into suitable attire; however, instead the person accepted but brought along her preferred attire, where she ultimately wore as per her likes.

*We had an official meeting and on those days, there is a dress code. So I consulted with the person so that she could dress officially. The person went and changed but carried her preferred attire and to my surprise, during the event, she was dressed in her preferred attire. I had nothing to say.* (IDI, Male Solid Waste Management Provider, Korogocho)

#### 3.1.3. Relationship perspective

Relationship perspectives include planning for PWDs based on their experiences, linking them with existing opportunities, and creating opportunities for PWDs at work, as the strategies enhance linkages and networking, hence low social conflict.

##### 3.1.3.1. Planning based on qualities, knowledge, and experience and linking with existing opportunities

Having asked questions and identified strengths, interests and choices, skills and qualities, and possible obstacles, the support network/staff members planned for participation by the PWD. Employers and support employees also researched opportunities in the wider community and included groups, facilities, businesses, or programs, and as such they remained alert for useful contacts, encounters, or information that may lead to suitable options for PWDs. Planning and linking with opportunities informed each other and often happened simultaneously; on the other hand, sometimes opportunities occurred spontaneously and became a basis for further planning. These were for additional skills that could not be offered by the employer and support groups on the job. There were also reports relating to lessons learnt by PWDs from a certain program on how to deal with unexpected everyday situations, but the program only run for a very short period. Despite that the employers offered on-the-job skills on how to deal with unexpected situations, they acknowledged the need of not abruptly disconnecting the employed person with their previous support, until the PWD was comfortable. As such they allowed the person to continue with their previous programs (i.e., programs before they joined the employer) on some days of the week, until the person considered the support from work as adequate.

*One person came to me very early at my office to report a program which was closed that helped deal with unexpected life situations. I had to call other employees who were supporting the PWD at work and we thought about how to connect the person with other programs. We connected the PWD to a program and he did not benefit … we then referred him to a community program, where they could attend for two hours in a week and he benefitted … but with time, the person realized the support at work is adequate for them.* (IDI, Female Education Provider, Korogocho)

Employers and employee support teams evaluated opportunities by considering factors such as geography, public transport, cost, and accessibility. Complex obstacles were worked through with a thought, persistence, and creativity. Risk came with independence, and this was a concern for network members balancing participation and protection. As such, there was a need for complex obstacles to be addressed by affirmative action office at the county. The initiatives were personal to employers and management teams of the different sectors in the attempt to support the most vulnerable in the community and sometimes in the attempt to meet the policy action of inclusivity at the workplace.

*We do all we can to resolve issues that affect persons with disability at work.* (IDI, Male Sub-county official, Korogocho)

##### 3.1.3.2. Creating targeted opportunities and facilitating access to opportunities

Employers and support groups established programs, groups, organizations, and facilities or sometimes expanded an existing opportunity for fitness to all including fitness to employed PWDs. Opportunities created by employers included soccer days, netball competitions, vocational education, exhibitions, book clubs, personal care support, securing furniture and appliances, health review, and community gardening, among other opportunities.

*We used to send the persons to* {Name of a program} *but it was not convenient. We started our own safe space where we have sports, care services, art, competitions, clubs and all sorts of opportunities. It is a small space but it has done wonders in supporting the disadvantaged people in the community … We talked to everyone to support our safe space so that everyone can access it. We've got churches on the side, clubs, local government, you name it … nothing ever in isolation and our groups have benefitted.* (IDI, Male Education Provider, Viwandani)

### 3.2. Theme 2: challenges in social support

Study participants described how misdirected and inadequate resources, dissatisfaction and unhappiness, and conflicts were constraints to social support to PWDs in the workplace.

#### 3.2.1. Misdirected and inadequate resources

The most commonly reported reason for the failure of support was the inadequate and often misdirected contribution of some support groups. Some groups frequently failed to identify what was meaningful to the person as well as opportunities and suitable strategies to enhance participation, taking a “one-size-fits-all” approach. Some support services seemed hampered by a lack of funding and staffing, hence the need to address most pressing needs, inaccurate beliefs, and inflexibility of policy both at a personal level and an organizational level, resulting in an inefficient use of resources to support PWDs.

*Due to inadequate funding or few qualified workers, employers find themselves in a situation where there is no real support for PWD or the support is channeled to most pressing issues and individualized support is lost.* (IDI, Female Sub-county official, Viwandani)

#### 3.2.2. Dissatisfaction and unhappiness

Sometimes the PWD communicated unhappiness to their job support group, or support groups reported dissatisfaction in the way the PWD performs their role. Notably, sometimes the support collapsed altogether. One person lost a job when a support group was not satisfied and unhappy because they were afraid that the person might jump out of the lorry without warning and hurt themselves.

*Feedback is key. One time a driver reported that the person he was supporting was about to jump from the lorry several times while transporting solid waste to the dumping site. The person lost the prestigious job because the driver was not happy and did not want him to lose his life while at work.* (IDI, Male Solid waste management Provider, Korogocho)

#### 3.2.3. Conflicts

Sometimes, employers and employees were in conflicts. Some conflicts stemmed from the disagreement, anger, closeness, and persistence in the workplace by the employed PWD with the employees in a support group. Remarkably, this could result in anger by the support group and a lack of willingness to offer maximum support. When conflicts had not always been anticipated, it could take months or years to negotiate, thus affecting social support offered to PWDs by the employee's or employer's network.

*There are conflicts of course between the support group of employees with the employed person with disability and this mostly results in anger and unwillingness to support … the conflicts could take long to be resolved if they are not anticipated.* (IDI, Male Sanitation Provider, Viwandani)

### 3.3. Theme 3: maintenance of the social support

From the employers’ perspectives, essential to the functioning and an enabling environment for PWDs were communication, coordination, sharing of the workload, and support by a network/support group. Employers did not randomly hire PWDs to make their life better; for some, there was a deliberate policy and action plan that necessitated the actions of engaging and supporting PWDs. For many, they employed PWDs to utilize their talents.

#### 3.3.1. Communication

Employers pooled information about PWDs and available opportunities. Information was made accessible, and all leads were followed to arrive at successful support strategies. This was often established at the beginning of work engagement and continued as the PWD was already involved at work.

*The PWD who was employed was introduced to diarizing progress by the employer. Employers could then produce a weekly or monthly summary of the changes in person. A close employer working with the PWD also summarized and shared on the progress report of the PWD.* (IDI, Male Education Provider, Korogocho)

#### 3.3.2. Coordination at work

Employees coordinated times and dates for meetings when extra support would be required by any employee, with a focus on PWDs. Records were kept, for example, meeting minutes or a goal chart for supporting PWDs.

*There are meetings coordinated to support employee in need, more so PWD. Minutes, action plans, and any emerging issues were used to resolve work related challenges.* (IDI, Male Solid Waste Management Provider, Viwandani)

#### 3.3.3. Sharing the workload

Support tasks and roles involved time and hard work, and as such support for PWDs was deliberately delegated. This was overwhelming for some network members; therefore, the employers were supportive in ensuring the PWD finds the right support when needed.

*‘The management supports a PWD achieve their goals … .It is usually who can help the person best. When? How? So we are deliberate with support, otherwise, it all becomes very wishy-washy and nobody knows who is going to do what.* (IDI, Male Education Provider, Korogocho)

#### 3.3.4. Supporting individual members

All employees inclusive of those without disability were appreciative of each other's time and input, were aware of personal circumstances, and offered support and understanding to each other when needed. When circumstances affected employees “contribution, avenues of communication remained open in the hope that support would be provided to anyone in need.”

*The employees without disability are dedicated to support PWD when needed. There are open avenues for communication when one needs support. Sometimes they think about it as if it were their family members.* (IDI, Female Water Provider, Korogocho)

## 4. Discussion

Social support identified in the study was in the form of listening to PWDs with a concern; identifying strengths and obstacles of the person; planning for the person based on qualities, knowledge, and experience and linking them with existing opportunities; creating opportunities and facilitating access to opportunities; gradual withdrawal of support by a support team; and compromise by a support team. This could be attributed to the fact that the National Council for Persons with Disabilities of Kenya has an array of commitments for PWDs and their environs, with concerns on way, drive, and engagement compared to other counties in LMICs (9). More often than not, employers seek to hire people who are highly productive, competent, and skillful at work. However, a few employers have gone beyond work centeredness to employ and provide social support for PWDs to enhance their productivity and capabilities. This is contrary to the traditional beliefs and perceptions that associate disability with ill health and depict PWDs as “sickly” and entities of misfortune, who should rely on handouts (18). Despite that for-profit firms–service providers may have additional pressures that may influence their attitudes and willingness to offer support for PWDs, employers who were interviewed capitalized on strengths and talents of PWDs for mutual benefits. We add to the literature that “begging” is the only available work option to PWDs, due to lack of willingness and ready employers, especially at the lower rungs of the employment spectrum. Our findings sought to add to the existing literature on PWDs in the labor market from the perspectives of employers. A review to explore studies on the employment of PWDs by the Organisation Economic Cooperation and Development (OECD) and other key actors discloses a worthy research on involvement (or lack thereof) of PWDs, with studies primarily concentrated on employees, and to the best of our knowledge, no specific studies in informal settlements approached the employment and inclusion matters from employers’ perspective (24, 40). Worthy to note is that Kenya has made some strides and has been portrayed in this study with employers and entrepreneurs offering social support to employed PWDs.

Beyond reported challenges faced by PWDs relating to negative treatment and associated psychological problems of self-esteem and other stressors in the community and at work, our study, guided by the social support theory, especially the postulation that social resources can be relied upon by individuals when dealing with lived challenges, realities, and stressors (27), builds on the need to understand the pathways through which PWDs will obtain the social resources they need for their effective functioning in their workplace. We sought to document what it will take to socially support an employed PWD from the perspectives of employers and entrepreneurs. Our study highlighted the best practices in the employment of PWDs, adding to an understanding of social support for PWDs in the labor market. There is evidence that not all employers and entrepreneurs have invested in the vision of inclusion of PWDs in the labor market (7, 41), despite the significant number of commitments made around social protection (4). This could be ascribed to many commitments relating to inclusion of PWDs that are fairly not precise and often broad (40). Thus, the commitments possibly engender reservations regarding what precisely is needed or a possibility that there is more evidence needed before such commitments are implemented (9). Particularly, employers and entrepreneurs could learn from the social support approaches identified and take precautions against the challenges pointed out in this study. Notwithstanding the scanty literature on social support for employed PWDs in low-income areas and the need to advocate for support, the commitments are broad and non-specific (9, 24), calling for more champions to support PWDs in the workplace. Further, results depict how disability is no longer relegated to charity in low-income areas but an inclusion issue, and some employers are championing this movement in Kenya by going beyond what the government expects in terms of quotas and incentives, by enhancing social support of PWDs, which is beyond the targets set by the government (20). The findings substantially differ from those of many previous studies (5, 42) describing how employers are reluctant in employing PWDs (41), in that the study paints a more realistic picture of the concerns and social support of employers toward employed PWDs in low-income areas.

## 5. Implications

Our results have implications on employment policies for PWDs and practice related to social support concerning PWDs in Kenya, more so in the informal settlements. Actors and institutions focusing on the welfare of PWDs could campaign for the execution and administration of disability laws documented in the Persons with Disabilities Act (43). The act forbids discrimination and necessitates that 5% of the contractual and spontaneous jobs be earmarked for PWDs and should be obligatory. This could be built on our findings that advance documenting best practices for enabling and inclusive work environments to maximize the productivity of people with disability in the workplace. Our study was driven beyond the “supply side” of the employment equation, to cover the “demand side” of the employment equation. Our intention here is to add value to the demand side of the employment of PWD equation by engaging a dominant missing link from the voices of employers and entrepreneurs themselves. Social support strategies are substantial for policy strategies that could reduce employer concerns about lawsuits or discrimination complaints after hiring workers with disabilities. Notably, social support strategies may slightly differ for employees with disability in other formal settings.

The findings have concrete implications for managing PWDs in the labor market. First, it is important to socially support workers with disabilities. As such, employers, employees without disability, and other actors should consider calls for support from workers with disabilities in each aspect of their tasks and at every instant of professional development. Second, organizations should design some support teams/groups at work according to definite levels of support required by workers with disabilities. Notably, establishing worker–organization feedback mechanisms would be key in building and fostering employer–employee work relations for suitable support of PWDs to fit into their tasks at work and offers the right balance between support demanded and support offered. Further, from our findings, distancing and withdrawal of some support to PWDs overtime could be a managing scheme for workers with disability to achieve independence, confidence, and sensitivity and be less vulnerable to work-related challenges (7, 18). Third, our findings could be beneficial to PWDs as it aids in designing and redesigning jobs to enable strengthening and inspiring of PWDs to self-sufficiency, personal growth, job empowerment, and reducing underemployment and discrimination in the workplace (24, 44). The findings also imply that PWDs will fit into their tasks at work, if social support at the workplace is designed to address their real needs as employees of normal organizations (18, 44).

The result might lead to a more diverse and accepting workplace for all employees: a more flexible approach to retaining skilled workers and hiring new employees, opportunities to increase productivity and take advantage of untapped talent, and a greater focus on job skills and performance rather than fear of potential future problems. Bringing in external experts to help with disability and accommodation issues could not only offer a broader range of solutions but also demonstrate good faith and ensure fair treatment and therefore potentially reduce legal liability. Employers could take a greater role in acquiring and centralizing the necessary information and expertise to better understand disability, appreciate workers’ abilities, and solve accommodation problems. They could also create company-wide procedures, policies, and mechanisms to place less responsibility and burden on the individual.

Our findings do not map the experiences of PWDs entering the workforce and in the workplace. However, the results captured perspectives of social support that might be expanded in future research to uncover the processes of joining workforce by PWDs.

## 6. Conclusion

Inclusion of PWDs includes crafting an all-encompassing workstation, where employed persons with disability are contented, appreciated, respected, and treasured at all times without judgement. Nobody wishes to be endured at work, and like those without disabilities, PWDs need to be acknowledged for their actions. Often, there is some skepticism and reservation that comes with a resolution to employ a PWD, as they are reflected to be a liability by many employers. Therefore, the primary objective of inclusion is to embrace competencies, skills, capabilities, and strengths of all, in diverse work settings without judgement. These will ensure that workplaces align to building an all-inclusive work environment for all. Notably, in the absence of jobs for everyone and high unemployment rates among every segment of the population, there is a need for a thorough national awareness operation in Kenya to mobilize and sensitize employers and investors around the competencies of PWDs and their need to socially support on an impartial basis. On the other hand, employment centers may consider setting up at various locations to advise and support PWDs on the job and career opportunities that are disability-friendly. Additionally, vocational centers should partner with employers and avail information about capabilities and skills of PWDs. On the other hand, the government should provide tax-related benefits to employers to upsurge employer incentives for hiring PWDs. Employment matters affecting PWDs are complex and require multi-pronged context-specific approaches. Thus, employers of PWDs and their supporters may need to be hands-on and involve diverse stakeholders to implement current policies, frameworks, and guidelines in different work contexts across the country and beyond. Worthy to note is that some PWDs have already internalized misconstructions and misapprehensions regarding their capabilities. As such, actions may be required to upsurge self-confidence and self-esteem in them.

The big challenge in labor market is how to get employers interested in social support, by being committed to changing their culture of taking employed PWDs as a liability. If the employment prospects of PWDs are to be significantly improved, key actors in the labor must pay attention to the ways in which corporate culture creates or reinforces obstacles for employees with disabilities and how those obstacles can be removed or overcome. The removal of barriers has significant benefits not just for PWDs but also for other employees and the organization as a whole. Experts in law, economics, human resources, regulatory compliance, corporate anthropology, disability studies and policy, and PWDs themselves need to collaborate in formulating a blueprint for the future study of disability and corporate culture. These results also point to the value of further research into employer behavior regarding employees with disability. It would be valuable to extend this research in the future, particularly because unlike the profiles constructed here, many PWDs in low-income areas are not employed, and when employed, they usually lack some social support. Further research could also assess the types of social information in hiring behavior, which can shed light on the reasons for lower interest in applicants with disabilities along with specific policies or practices that can reduce this problem. For example, how do employers react when they are confronted with an application from a PWD, what are the steps in their reaction, and what are the beliefs, attitudes, and hiring behaviors? What is the role of written disability policies, training, and support from management? Such research can help identify the most effective policies and practices to increase their employment opportunities and social support for employed PWDs.

PWDs, employers with PWDs, and labor activists should endeavor to engage in policy dialog to raise awareness of the barriers faced by people with disabilities and the opportunities to strengthen their social support in employment; support employers in implementing disability-inclusive policies, practices, and services; and support research and data collection in low-income areas to understand the key needs of and barriers faced by PWDs regarding employment, so as to establish examples of good practice and lead by example in providing support to employees with disabilities. Policymakers should endeavor to review national labor laws to ensure that they do not create unintended disincentives to the employment of people with disabilities; codify minimum standards for accessibility in law, while allowing for flexible and incremental implementation of guidelines supporting employed PWDs; offer positive incentives for employers to employ people with disabilities; and equip public employment services to support jobseekers with disabilities. On the other hand, private sectors should introduce targeted recruitment and in-work support for PWDs, give PWDs opportunities to enhance their skills, and adopt or promote the best practice in the universal design of products and services for employed PWDs.

## Data Availability

The raw data supporting the conclusions of this article will be made available by the authors, without undue reservation. The request can be made through https://aphrc.org/microdata-portal/.
